# Enhancing salinity stress tolerance in lemongrass (*Cymbopogon citratus* L.) using foliar-applied silicon nanoparticles

**DOI:** 10.1186/s12870-026-09312-0

**Published:** 2026-06-30

**Authors:** Ahmed Mahdy, Nawra Marzouk, Mervat El-Hefny, Tarek El-keiy

**Affiliations:** 1 Department of Soil and Water Sciences, Faculty of Agriculture, Alexandria University, Alexandria, Egypt; 2 Department of Floriculture, Faculty of Agriculture, Ornamental Horticulture and Landscape Gardening, Alexandria University, Alexandria, Egypt

**Keywords:** Lemongrass, Cymbopogon citratus, Silicon nanoparticles, Foliar application, Salt tolerance, Oxidative stress, Medicinal plants, Plant growth regulation

## Abstract

Salinity stress is a major abiotic constraint limiting the growth, productivity, and essential oil quality of medicinal and aromatic plants, including lemongrass (*Cymbopogon citratus*). Therefore, this study was conducted during the 2022 and 2023 seasons to evaluate the effects of different salinity levels and silicon nanoparticle (SiNPs) applications on vegetative growth, rhizome characteristics, nutrient status, and essential oil yield and composition of lemongrass plants. The experiment aimed to (i) assess the impact of irrigation water salinity (0.7, 2, 4, and 6 dS m^-1^) on plant performance, (ii) investigate the role of SiNPs applied as a foliar (400, 800, and 1200 mg L^-1^) treatments in alleviating salinity stress, and (iii) determine the interaction effects between salinity levels and SiNPs on growth and oil productivity. The results revealed that low salinity levels (0.7 and 2 dS m^-1^) significantly enhanced vegetative growth parameters, including plant height, number of tillers and leaves, biomass accumulation, and leaf area, as well as rhizome traits and essential oil yield, compared with higher salinity levels. In contrast, increasing salinity (4 and 6 dS m^-1^) markedly reduced growth, nutrient uptake (N, P, and K), and oil productivity, while increasing proline accumulation. The application of SiNPs, particularly at 1200 mg L^-1^ (foliar), significantly mitigated the adverse effects of salinity by improving growth traits, enhancing photosynthetic efficiency, and promoting nutrient uptake. Moreover, SiNPs positively influenced essential oil yield and modified its chemical composition, increasing key constituents such as citral and related compounds under stress conditions. In conclusion, silicon nanoparticles effectively enhanced lemongrass tolerance to salinity stress and improved both yield and quality across both growing seasons. The combined application of appropriate salinity management and SiNPs represents a promising strategy for sustainable cultivation of lemongrass under saline environments.

## Introduction

Salinity stress is one of the most severe abiotic constraints limiting agricultural productivity worldwide, affecting a substantial proportion of arable and irrigated lands. It disrupts plant growth by inducing osmotic stress, ion toxicity, and oxidative damage, ultimately impairing physiological and metabolic processes such as photosynthesis, nutrient uptake, and enzyme activity. In aromatic and medicinal plants such as *Cymbopogon citratus* L. (lemongrass), salinity not only reduces biomass production but also negatively affects essential oil yield and quality, thereby diminishing its economic value.

In recent years, nanotechnology has emerged as a promising and sustainable approach to enhance plant tolerance to environmental stresses. Among various nanomaterials, silicon nanoparticles (SiNPs) have gained considerable attention due to their unique physicochemical properties, high reactivity, and efficient uptake by plant tissues. Accumulating evidence suggests that SiNPs play a crucial role in mitigating abiotic stresses by improving plant growth, enhancing nutrient use efficiency, and regulating physiological and biochemical processes [1]. Compared to conventional silicon sources, nano-silicon exhibits greater bioavailability and effectiveness, particularly when applied through foliar spraying.

Foliar application of silicon nanoparticles has been reported as an efficient delivery method that directly supplies silicon to aerial plant parts, enhancing rapid absorption and utilization. Recent studies have demonstrated that foliar-applied SiNPs significantly alleviate salinity-induced damage by improving photosynthetic performance, maintaining water balance, and regulating ion homeostasis. Moreover, SiNPs enhance antioxidant defense systems by stimulating key enzymes such as superoxide dismutase, catalase, and peroxidase, thereby reducing oxidative stress and membrane damage under saline conditions [2]. Additionally, nano-silicon contributes to osmotic adjustment and promotes the accumulation of compatible solutes, which further supports plant adaptation to salt stress.

In lemongrass and related species, the application of silicon nanoparticles has shown promising results in improving growth, physiological traits, and secondary metabolite production under salinity stress. For instance, foliar application of SiNPs enhanced plant height, biomass, photosynthetic efficiency, and essential oil content while reducing reactive oxygen species accumulation in salt-stressed plants [3]. These beneficial effects are attributed to the ability of silicon to modulate nutrient uptake, strengthen cellular structures, and improve overall stress resilience.

Despite these advances, studies focusing specifically on *Cymbopogon citratus* L. remain limited, particularly regarding the role of foliar-applied silicon nanoparticles under saline conditions. Therefore, investigating the effectiveness of SiNPs in mitigating salinity stress in lemongrass is essential to develop sustainable strategies for improving productivity and quality in salt-affected environments. The present study aims to evaluate the role of foliar-applied silicon nanoparticles in enhancing salinity stress tolerance in *Cymbopogon citratus* L. plants. The specific objectives are: to synthesize and characterize silicon nanoparticles that bio-extracted from rice husk; to evaluate the role of foliar-applied silicon nanoparticles in enhancing salinity stress tolerance in *Cymbopogon citratus* L. Specifically, to investigate the effects of nano-silicon on plant growth and biomass, physiological traits, antioxidant defense systems, ion homeostasis, osmoprotectant accumulation, and essential oil yield and quality under saline conditions. This study provides a comprehensive evaluation of foliar-applied silicon nanoparticles as an innovative strategy to mitigate salinity stress in *Cymbopogon citratus* L., integrating morphological, physiological, biochemical, and productivity-related responses. Unlike previous studies that primarily focused on conventional silicon sources or other plant species, this work specifically addresses the mechanistic role of nano-silicon in enhancing stress tolerance and essential oil productivity in lemongrass. The findings are expected to contribute to the development of sustainable nanotechnology-based approaches for improving crop performance in salt-affected environments.

## Materials and methods

### Experimental Layout

#### Location

The study was conducted over two successive summer seasons (2022 and 2023) at the Nursery of the Department of Floriculture, Ornamental Horticulture, and Landscape Gardening, Faculty of Agriculture, Alexandria University, Egypt. The area has a Mediterranean climate with hot, dry summers. Mean monthly temperatures, average humidity, and wind speed during both seasons are presented in Table 1.

**Table 1:** Meteorological data of the experimental location during the 2022 and 2023 growing seasons

**Month**	**Max. Temp. (°F)**		**Min. Temp. (°F)**		**Humidity (%)**		**Wind Speed (mph)**	
	**2022**	**2023**	**2022**	**2023**	**2022**	**2023**	**2022**	**2023**
April	78.67	76.61	50.60	56.00	64.18	64.10	7.91	8.84
May	80.00	80.84	59.29	61.58	66.69	66.38	7.96	9.62
June	85.93	86.07	67.73	68.80	68.18	68.44	8.03	8.46
July	87.06	91.42	70.03	74.71	66.82	68.69	8.52	9.02
August	89.52	90.58	71.00	73.35	64.95	65.76	7.15	7.48
September	89.37	90.37	69.30	70.13	55.56	65.70	8.09	7.80
October	81.40	82.71	66.23	66.77	54.89	69.11	7.40	6.74

The meteorological data indicated generally similar climatic conditions between the two seasons, with slightly higher temperatures recorded in 2023, particularly during July and August. Relative humidity showed more variability in 2023, especially in September and October, while wind speed exhibited minor fluctuations between the two seasons.

### Synthesis and characterization of silicon nanoparticles (SiNPs)

Silicon nanoparticles were synthesized from rice husks obtained from rice mills in Damanhur City, El Behaira Governorate, Egypt, following the method of Alshatwi et al. [4]. Briefly, 20 g of rice husk was mixed with 100 mL of 1 N HCl and stirred using a magnetic stirrer. The mixture was autoclaved at 120 °C for two hours, washed with deionized water to remove residual acid, and dried at 85 °C for five hours until the black residue turned white. The synthesized SiNPs were characterized using scanning electron microscopy (SEM) and energy-dispersive X-ray (EDX) at the Central Laboratory, Faculty of Science, Alexandria University, Egypt, while crystallinity was analyzed by X-ray diffraction (XRD) at the College of Higher Studies for Nanotechnology, El-Sheikh Zayed City, Cairo, Egypt.

### Experimental treatments – foliar application

Uniform lemongrass (*Cymbopogon citratus* L.) tillers were obtained from the Nursery of the Department of Floriculture, Alexandria University. Rooted tillers (≈15 cm in length) were transplanted into 30 cm diameter and 30 cm height clay pots containing clay loam soil on April 15, 2022 and 2023. Plants were irrigated with tap water for one month to reduce transplanting stress before introducing salinity treatments.

Salinity treatments included four levels: 0.7 (control, tap water), 2, 4, and 6 dSm^-1^, prepared by diluting seawater (55 dSm^-1^) with tap water, the EC of blended water was measured to verify the final salinity level according to the following formula:

## Salt concentration OR EC in blended water = (C_A_​ × P_A_​) + (CB​ × PB​)

Where:

- C_A_​ = Salt concentration OR EC in Water A
- C_B_​ = Salt concentration OR EC in Water B
- P_A_​ = Fraction (or percentage) of Water A in the mixture
- P_B_​ = Fraction (or percentage) of Water B in the mixture

with: P_A_ + P_B_ = 1

SiNPs levels were 0, 400,800, and 1200 mg/L as foliar applied. Each SiNPs level was sprayed at four doses in sunset to prevent the leaves from sunburn. Foliar application continued until the plants were well treated with the solution. The first spray was applied one month after transplanting, the second after 21 days, the third one month after the first harvest, and the fourth after 21 days. All sprays included 0.01% Tween 20 as a surfactant [5]. Plants were harvested twice per season (August and October) by cutting the vegetative parts 20 cm above soil level.

The experiment followed a Randomized Complete Block Design (RCBD). Each block represents one salinity level and 4 levels of SiNPs with three replicates with the total 12 pots per block. The same treatments were done for the other salinity levels with three replicates. The total numbers of pots for all treatments were 48 pots as follows:

- Salinity levels (4) x SiNPs levels (4) x Replicates (3) = 48 pots

Each replicate included one plant per pot. The average was calculated for the three replicates (three plants/three pots). Soil physical and chemical properties are provided in Table 2, while seawater composition is in Table 3.

**Table 2:** Some physical and chemical characteristics of the studied soil

Soil physical properties						
**Clay (%)**		**Silt (%)**		**Sand (%)**		**Soil Texture**
42.00		18.90		40.60		Clay loam

**Soil chemical properties**						
**EC (dS m**^**-1**^**)**	**pH**	**CaCO₃ (%)**	**N (mg/kg)**	**P (mg/kg)**	**K (mg/kg)**	
1.70	8.47	8.55	42.00	15.90	469.50	

**Soluble cations and anions (meq L**^**-1**^**)**						
**Na**^**+**^	**Mg**^**2+**^	**K**^**+**^	**Ca**^**2+**^	**SO**_**4**_^**2-**^	**HCO**_**3**_^**-**^	**Cl**^**-**^
6.90	3.00	3.50	4.00	9.00	2.00	6.00

**Table 3:** Chemical analysis of seawater used in the study for salinity levels preparations

**Sample**	**Salinity**	**pH**	**Ca**^**2+**^	**Mg**^**2+**^	**Na**^**+**^	**K**^**+**^	**HCO**_**3**_^**-**^	**Cl**^**-**^	**SO**_**4**_^**2-**^	**SAR**
(mg/L)										
Seawater	45,120	7.8	132.00	189.00	380.00	6.00	135.00	368.00	200.00	30.00

The soil was classified as clay loam with moderate electrical conductivity (1.70 dSm^-1^) and alkaline pH (8.47), indicating slightly saline and calcareous conditions. Macronutrient levels revealed adequate potassium but relatively moderate nitrogen and phosphorus availability. The seawater analysis showed very high salinity (45,120 mg/L) with dominant sodium and chloride ions, reflecting its suitability as a strong salinity stress treatment.

### Vegetative growth and biochemical analyses

#### Vegetative growth parameters

After each harvest, the following growth traits were measured for each plant:

- Plant height (cm): Distance from soil surface to the tip of the tallest leaf.
- Number of tillers and leaves per plant: Counted and averaged per treatment.
- Leaf fresh and dry weight (g): Measured immediately after harvest; dry weight obtained after oven-drying at 70 °C to constant weight.
- Leaf length (cm) and area (cm^2^): Leaf area calculated as:
  - Leaf area = maximum length × maximum width × 0.75 [6].
- Rhizome traits: Diameter (mm), length (cm), fresh weight (g), and dry weight (g) were recorded for each plant.

#### Physiological and biochemical parameters

- Chlorophyll Value Index: Determined using a SPAD meter on fresh leaves [7].
- N, P, and K Content: Dried leaves were ground and digested in H_2_SO_4_ and H_2_O_2_. Total nitrogen was measured by Kjeldahl method [8], phosphorus by vanadomolybdate-yellow method at 470 nm [9], and potassium by flame photometry [9].
- Proline Content (µg/g): Determined following Bates et al. [10]. Fresh leaves (0.5 g) were homogenized in 30% sulfosalicylic acid, reacted with acid ninhydrin and glacial acetic acid, incubated at 100 °C, extracted with toluene, and absorbance read at 520 nm.

#### Essential oil extraction and analysis

- Oil extraction was done using fresh leaves (100 g) and hydro-distilled in a Clevenger apparatus for 2 h according to the methods of Taha et al., [11].
- Oil Percentage (%): Calculated as:
  - Essential oil % = (Oil volume/Fresh herb weight) × 100 [12].
- Oil Yield/Plant: Oil yield = Oil % × Fresh herb weight per plant.
- GC–MS Analysis: Chemical composition of essential oils was analyzed using Trace GC Ultra-ISQ with TG–5MS column. Identification was based on WILEY 09 and NIST 14 databases, using a match factor ≥ 650 [11].

The experiment was conducted as a two-factor factorial in a Randomized Complete Block Design (RCBD) with three replicates. The factors included Salinity levels and Foliar-applied SiNP doses. Each replicate included three pots per treatment, with one plant per pot. Data from the three pots were averaged to represent a single experimental unit.

### Statistical analysis

All data were analyzed using SAS software [13]. Factorial analysis for the 2-factor RCBD was performed, and treatment means were compared using LSD at 0.05 probability level [14]. Data were presented as the mean ± standard error of three replicates.

## Results and discussions

### Characterization of synthesized silicon nanoparticles (SiNPs)

The synthesized silicon nanoparticles (SiNPs) were comprehensively characterized using SEM, TEM, EDX, FTIR, and XRD to assess their morphology, elemental composition, surface functional groups, and crystallographic structure (Table 4). These analyses collectively provide insight into the structural and physicochemical properties of the nanoparticles.

**Table 4:** The characterization of the synthesized silicon nanoparticles (SiNPs) used in the study

Property	This Study (Synthesized SiNPs)	Literature Values/References	Notes
Particle Size (SEM/TEM)	Uniform particles, 1–100 nm	10–200 nm commonly reported (Chandrasekar et al., 2013) [15]	Matches typical nanosilica ranges; good control of morphology
Particle Shape	Spherical/near-uniform morphology	Spherical or quasi-spherical (Alshatwi et al., 2015) [4]	Typical for silica formed through hydrolysis/condensation methods
Surface Elemental Composition (EDX)	Si, O, C; trace Fe, P, Cu, Co, Cr	Si, O dominant; minor C; occasional trace metals (natural-source silica studies)	Trace metals fall within expected levels for environmentally derived precursors
O–H Vibration (FTIR)	Strong band at 3473 cm^-1^	3400–3500 cm^-1^ (Alshatwi et al., 2015) [4]	Indicates surface hydroxyl groups (silanol groups)
C = O Vibration (FTIR)	Band at 1644 cm^-1^	1630–1660 cm^-1^ (Alshatwi et al., 2015) [4]	Often due to adsorbed water or residual organics
Si–O–Si Asymmetric Stretching (FTIR)	1101 cm^-1^	1080–1120 cm^-1^ (Alshatwi et al., 2015) [4]	Typical fingerprint region for silica
Si–O–Si Symmetric Stretching (FTIR)	805 cm^-1^	780–820 cm^-1^ (Alshatwi et al., 2015) [4]	Confirms silica network formation
Si–O Bending (FTIR)	488 cm^-1^	450–500 cm^-1^	Matches classical vibrational mode of amorphous silica
XRD Pattern	Broad peak at ~ 21° and weak feature near 43° (amorphous)	Broad peak at 2θ = 20–25° (amorphous silica)	Confirms absence of crystalline silicon; typical for amorphous nanosilica
Crystallinity	Amorphous	Primarily amorphous for sol–gel and plant-derived SiNPs	Amorphous forms show higher reactivity and better adsorption
Purity	High purity silica nanoparticles	High purity silica commonly reported in controlled synthesis methods	Consistent with literature quality benchmarks

SEM and TEM images revealed well-defined, uniformly shaped nanoparticles with smooth surfaces and no significant agglomeration, indicating excellent dispersibility (Table 4). Particle sizes consistently ranged within 1–100 nm, demonstrating that the adopted synthesis method efficiently controlled nucleation and growth, yielding nanoparticles of high physical homogeneity.

EDX analysis confirmed that silicon and oxygen were the primary components of the nanoparticles, consistent with the formation of a silica layer surrounding the Si core (Table 4). Minor carbon content likely originated from residual organic matter or surface-stabilizing molecules. Trace amounts of iron, phosphorus, copper, cobalt, and chromium were also detected, probably deriving from the natural rice husk precursor or processing equipment, as commonly reported in biogenic silicon nanoparticles [15]. EDX analysis detected trace amounts of Fe, P, Cu, Co, and Cr in the synthesized SiNPs. These elements were present only as minor constituents and likely originated from the plant-derived precursor material or natural impurities associated with the synthesis process. The low abundance of Cu and Cr suggests that their concentrations were well below phytotoxic levels and therefore unlikely to pose risks to plant growth under the experimental conditions. Consistent with this interpretation, no toxicity symptoms or growth suppression attributable to heavy metal contamination were observed. However, future studies employing quantitative elemental analysis and long-term environmental monitoring are recommended to further evaluate the safety profile of the synthesized nanoparticles.

FTIR spectra identified multiple surface functional groups (Table 4). A broad band at 3473 cm^-1^ was assigned to O–H stretching, indicating hydroxyl or silanol groups, while the band at 1644 cm^-1^ corresponded to C = O stretching from residual organics or adsorbed CO_2_. Strong absorption at 1101 cm^-1^ indicated Si–O–Si asymmetric stretching, and peaks at 805 cm^-1^ and 488 cm^-1^ were attributed to Si–O symmetric stretching and bending vibrations, respectively [4, 16, 17]. These features confirm the presence of a well-developed amorphous silica network with chemically active surface sites.

XRD patterns showed a broad peak at 2θ ≈ 21° with a less pronounced feature near 43° (Table 4), characteristic of amorphous silica. The absence of sharp diffraction peaks indicates lack of long-range crystalline order, consistent with surface oxidation during synthesis that forms silicon oxide layers.

Overall, the comprehensive characterization of the synthesized silicon nanoparticles (SiNPs) offers important insights into their structural, chemical, and crystallographic features, which collectively influence their suitability for environmental, agricultural, and industrial applications. The characterization results confirm that the synthesized SiNPs are predominantly amorphous, uniformly distributed, and chemically stable silica nanoparticles with abundant surface hydroxyl groups.

These attributes make them highly suitable for various applications, including heavy metal adsorption, water purification, soil nutrient delivery, and catalytic processes. The combination of controlled particle size, structural stability, and reactive surface functionality highlights their strong potential in environmental and agricultural technologies.

### Effect of foliar-applied silicon nanoparticles under salinity stress on vegetative growth of *Cymbopogon citratus*

Vegetative growth parameters analyzed included plant height, number of tillers per plant, number of leaves per plant, fresh and dry leaf weight, leaf length, and leaf area per plant.

Analysis of variance (Table 5) for each parameter showed that salinity, SiNPs levels, and their interactions significantly influenced most parameters across both cuts and seasons, except for plant height and leaf length in the second season, and leaf area in the first cut of the first season. In the second cut, salinity affected all traits except leaf dry weight in the second season, while SiNPs application significantly influenced all traits except leaf dry weight in both seasons. Two-factor interactions were generally significant, except for leaf dry weight in the second season.

**Table 5:** Analysis of variance for vegetative characters (plant height, number of tillers per plant, number of leaves per plant, leaves fresh weight per plant, leaves dry weight per plant, leaf length and leaf area per plant) in the two consecutive seasons of 2022 and 2023

First cut															
**Source of variation**	**Degree of freedom**	**plant height (cm)**		**No. of tillers/plant**		**No. of leaves/plant**		**leaves fresh weight/plant(g)**		**leaves dry weight/plant (g)**		**leaf length (cm)**		**leaf area/plant (cm**^**2**^**)**	
		** 1 st season**	**2nd season**	** 1 st season**	**2nd season**	** 1 st season**	**2nd season**	** 1 st season**	**2nd season**	** 1 st season**	**2nd season**	** 1 st season**	**2nd season**	** 1 st season**	**2nd season**
Rep	2	0.636^¶^	1.83	0.043	0.206*	2.46	3.41	2.72	1.49	7.14	17.20	1.69	0.111	20,107.04	8530.32
Salinity (A)	3	22.46*	22.61**	1.50**	2.036**	35.88**	26.58**	834.41**	76.79**	559.57**	241.85**	44.86**	28.32**	510,833.95**	246,028.02**
Silicon (C)	3	207.36**	4.45	0.31*	0.707**	68.20**	24.77**	276.80**	54.84**	289.84**	288.18**	84.50**	3.30	646,914.29**	99,825.75**
A*C	9	34.09**	11.78**	1.18**	0.378**	10.42*	16.96**	94.66**	77.91**	84.05**	19.77*	15.33**	8.65**	46,195.00	64,454.08**
error	30	6.56	2.20	0.079	0.056	3.61	2.25	1.72	0.8207	20.29	6.71	1.24	1.36	21,470.17	10,745.10

Second cut															
Rep	2	1.92	49.90	0.176	0.287	8.53	4.13	39.10	29.75	113.36	1822.90	0.471	7.65	44,476.93	17,692.03
Salinity (A)	3	158.67**	380.81**	0.526**	2.14**	26.63**	261.55**	107.17**	700.47**	1032.07*	253.66	101.45**	580.93**	213,569.03**	3,823,351.47**
Silicon (C)	3	160.07**	31.59**	2.04**	1.51**	115.81**	25.81**	291.58**	57.13**	647.38	266.30	87.40**	21.39**	303,369.04**	298,688.95**
A*C	9	71.13**	28.26**	1.42**	2.96**	26.96**	58.29**	164.83**	387.83**	1092.41**	142.40	45.98**	29.17**	167,615.69**	349,366.14**
error	30	5.52	2.56	0.107	0.173	5.46	5.54	10.94	7.58	326.33	268.16	7.06	2.98	36,168.01	40,778.90

^*^, ^**^: significant at 0.05 and 0.01 probability levels, respectively

^¶^Data represent F-value

#### Plant height (cm)

Salinity stress significantly reduced the plant height of *Cymbopogon citratus* across both seasons and cuts (Table 6). In the 2022 season, the first cut plant height under control conditions decreased from 70.44 cm at 0 dSm^-1^ to 66.55 cm at 6 dSm^-1^ at zero SiNPs application rate, while the second cut ranged from 67.36 cm to 64.51 cm across the same salinity levels at zero SiNPs application rate, with differences exceeding the L.S.D (0.05) values for salinity (A = 2.13 cm for first cut, A = 1.95 cm for second cut), confirming significant reductions. Similarly, in 2023, the first cut plant height decreased from 55.32 cm to 53.55 cm and the second cut from 77.97 cm to 67.33 cm under control conditions at zero SiNPs application rate, with L.S.D (0.05) values of 1.23 cm and 1.33 cm, respectively, indicating statistically significant effects of salinity. Foliar application of silicon nanoparticles (SiNPs) markedly mitigated these reductions, with the highest concentration (1200 mgL^−1^) consistently producing the tallest plants across all salinity levels. For example, in 2022 first cut, plant height at 6 dSm^-1^ salinity level increased from 66.55 cm at 0 mg/L SiNPs to 81.66 cm at1 200 mgL^−1^SiNPs application rate, exceeding the interaction L.S.D (A × C = 4.27 cm), while in 2023 s cut, height increased from 67.33 cm to 72.69 cm at the same salinity (zero salinity level) and SiNPs (1200 mg/L) level (A × C = 2.67 cm). The positive effect of SiNPs was most pronounced under moderate salinity (2–4 dSm^-1^), where increases of 5–7 cm over untreated plants were observed. Seasonal and cut differences were also evident; second-cut plants in 2023 showed greater heights than first-cut plants under similar treatments, likely reflecting favorable climatic or management conditions. These results indicate that SiNPs enhance salinity tolerance by maintaining ionic balance, reducing Na^+^ toxicity, strengthening cell walls, and improving water use efficiency and photosynthetic performance. Overall, foliar application of SiNPs, particularly at 1200 mgL^−1^, significantly counteracts salinity-induced growth inhibition in lemongrass, providing a statistically validated and practical solution for improving productivity under saline conditions.

**Table 6:** Effect of foliar application of silicon nanoparticles (SiNPs) on plant height (cm) of Cymbopogon citratus plants grown under salt stress during the seasons of 2022 and 2023, both first and second cuts

**Season**	**Cut**	**Salinity (A) (dSm**^**-1**^**)**	**SiNPs levels(C) (mgL**^**−1**^**)**				
			**0**	**400**	**800**	**1200**	**Mean**
2022	1st	Control	70.44 bc	72.44 b	75.33 b	83.44 a	75.41 a
		2	68.99 bc	74.00 b	74.55 b	74.66 b	73.05 b
		4	66.77 c	73.10 b	75.55 b	76.21 b	72.91 b
		6	66.55 c	68.66 bc	72.33 b	81.66 a	72.30 b
		Mean	68.19 d	72.05 c	74.44 b	78.99 a	
		L.S.D _0.05_	A = 2.13 C = 2.13 A × C = 4.27				
	2nd	Control	67.36 d	74.59 b	76.36 b	80.29 a	74.58 a
		2	63.59 e	66.51 d	72.84 b	83.74 a	71.67 b
		4	67.70 d	68.36 d	72.40 b	75.88 b	71.09 b
		6	64.51 d	65.29 d	66.51 d	67.06 d	65.84 c
		Mean	65.79 d	68.69 c	72.03 b	76.74 a	
		L.S.D _0.05_	A = 1.95 C = 1.95 A × C = 3.92				
2023	1st	Control	55.32 b	57.10 b	59.21 a	60.77 a	58.10 a
		2	56.66 a	57.33 b	58.55 a	59.66 a	58.05 a
		4	56.22 b	56.55 b	57.77 b	59.99 a	57.63 a
		6	53.55 bc	54.55 bc	55.21 bc	57.55 b	55.21 b
		Mean	55.44	56.38	57.69	59.49	
		L.S.D _0.05_	A = 1.23 C = N.S A × C = 2.47				
	2nd	Control	77.97 c	83.05 ab	85.55 a	86.16 a	83.18 a
		2	72.11 de	75.22 cd	76.61 c	81.72 b	76.41 b
		4	70.28 e	72.22 de	73.83 d	74.88 cd	72.80 c
		6	67.33 f	70.33 e	70.44 e	72.69 d	70.20 d
		Mean	71.92 c	75.21 b	76.61 b	78.86 a	
		L.S.D _0.05_	A = 1.33 C = 1.33 A × C = 2.67				

#### Number of tillers per plant

**Fig. 1:**
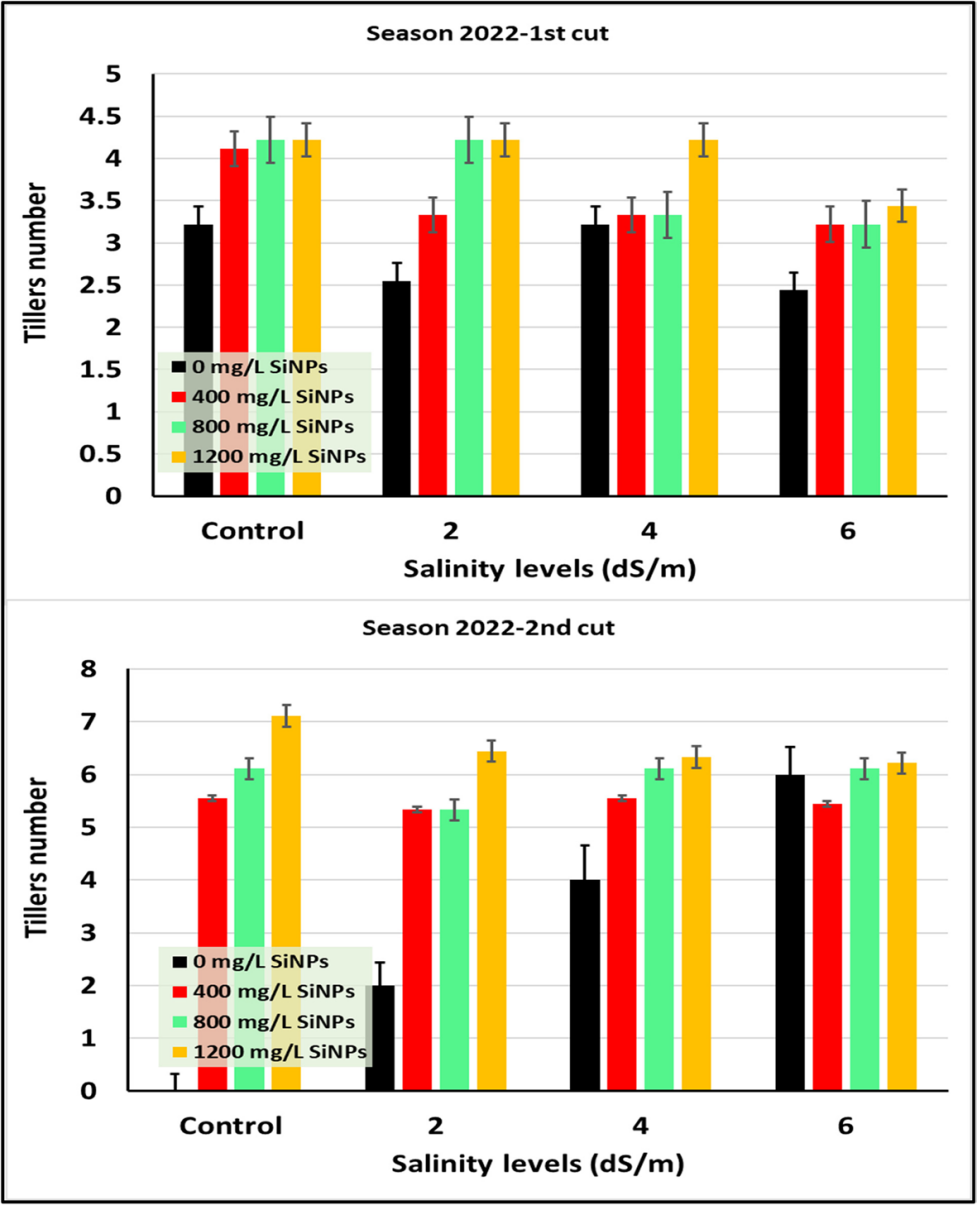
Effect of foliar application of silicon nanoparticles (SiNPs) on number of tillers of *Cymbopogon citratus* plants grown under salt stress during first and second cut of season 2022. Error bars represent standard error. Bars correspond to SiNPs concentrations (0, 400, 800, and 1200 mgL.^−1^)

**Fig. 2:**
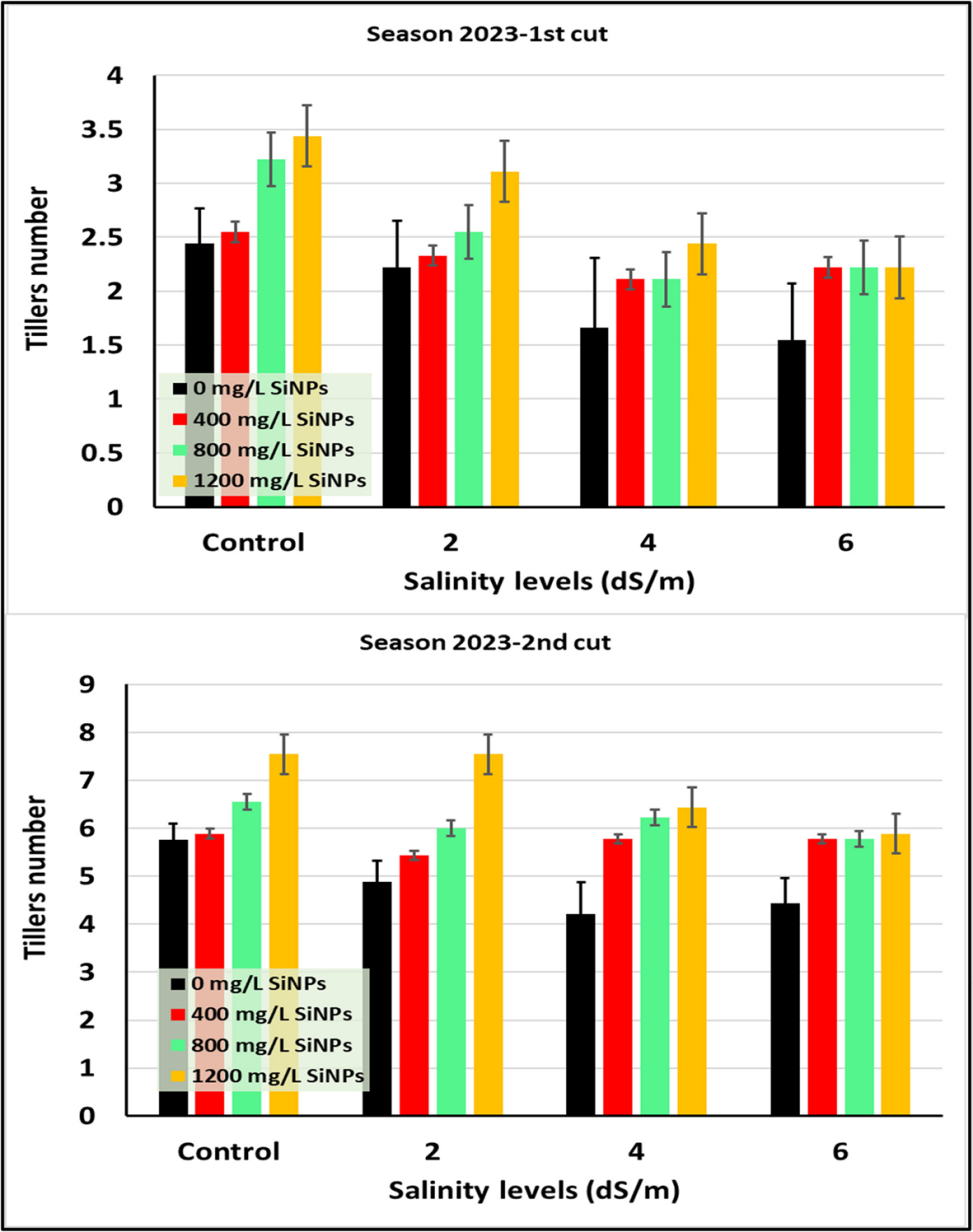
Effect of foliar application of silicon nanoparticles (SiNPs) on number of tillers of *Cymbopogon citratus* plants grown under salt stress during first and second cut of season 2023. Error bars represent standard error. Bars correspond to SiNPs concentrations (0, 400, 800, and 1200 mgL^−1^)

Salinity significantly reduced tiller numbers in both cuts and seasons (Figs. 1 and 2). Control plants (Under 0 dS/m + 0 SiNPs) consistently produced the highest number of tillers, while 6 dSm^-1^ resulted in the lowest counts. For example, for the first cut-2022, tiller number was 3.22, declining to 2.44 at 6 dS/m (LSD = 0.33).While, for the second cut-2022, tiller number was 5.55, declining to 5.44 at 6 dS/m (LSD = 0.11).Moreover, for the first cut-2023, tiller number was 2.44, declining to 1.55 at 6 dS/m (LSD = 0.23).While, for the second cut-2023, tiller number was 5.77, declining to 4.44 at 6 dS/m (LSD = 0.11).

Foliar SiNPs significantly enhanced tiller development across all salinity levels. In both cuts, 1200 mgL^−1^ SiNPs produced the maximum number of tillers, and interaction effects indicated that SiNPs application at moderate to high salinity restored tillering to near-control levels.

#### Number of leaves per plant

The number of leaves decreased with increasing salinity. In the first cut, control plants had the highest leaf numbers, whereas 6 dSm^-1^ showed the lowest values. Foliar SiNPs significantly increased leaf numbers across all treatments. Interaction analysis revealed that higher SiNPs doses (800–1200 mg/L) under 2–4 dSm^-1^ salinity restored leaf development to levels comparable with the non-saline control (Table 7).

**Table 7:** Effect of foliar application of silicon nanoparticles on the number of leaves/plant of *Cymbopogon citratus* plants grown under salt stress in the two consecutive seasons of 2022 and 2023

**Season 2022**					
First Cut					
Salinity levels (A) (dSm^-1^)	SiNPs levels (C), (mgL^-1^)				Mean
	0	400	800	1200	
Control	25.11 a	25.33 a	25.77 a	26.11 a	25.58 a
2	19.55 c	23.11 b	24.44 a	25.21 a	23.08 b
4	17.77 c	23.66 b	25.77 a	27.21 a	22.80 bc
6	16.88 c	21.07 b	22.99 b	24.77 a	21.43 c
Mean	19.83 b	23.29 a	24.74 a	25.83 a	
L.S.D _0.05_	A= 1.58	C= 1.58		A×C= 3.17	
Second Cut					
Control	24.44 bc	28.89 a	29.89 a	30.99 a	28.55 a
2	22.44 bc	23.33 bc	25.55 b	32.66 a	26.00 b
4	22.33 bc	23.89 bc	24.00 bc	33.33 a	25.88 b
6	21.55 c	23.33 bc	28.66 ab	27.00 b	25.13 b
Mean	22.69 d	24.86 c	27.03 b	30.99 a	
L.S.D _0.05_	A= 1.94	C= 1.94		A×C= 3.89	

Season 2023					
First Cut					
	0	400	800	1200	
Control	17.10 cd	20.88 b	24.88 a	25.66 a	22.13 a
2	19.66 bc	19.77 bc	21.32 b	21.88 b	20.66 b
4	17.44 c	18.00 bc	20.21 b	22.55 b	19.55 bc
6	16.99 d	17.55 c	19.55 bc	20.66 b	18.69 c
Mean	17.80 b	19.05 b	21.49 a	22.69 a	
L.S.D _0.05_	A= 1.25	C= 1.25		A×C= 2.50	
Second Cut					
Control	36.22 b	36.66 b	36.66 b	44.66 a	38.47 a
2	28.89 d	31.11 c	37.11 b	40.00 b	34.27 b
4	28.11 d	35.00 c	35.22 c	36.11 b	33.61 b
6	22.66 e	28.00 d	28.33 d	29.66 d	27.16 c
Mean	28.97c	32.69 b	34.33 b	37.61 a	
L.S.D _0.05_	A= 1.96	C= 1.96		A×C= 3.92	

Means followed by the same letter(s)are not significantly, at 0.05 level of probability

#### Fresh and dry leaf weight

Salinity negatively affected both fresh and dry leaf weight. The greatest reduction occurred under 6 dSm^-1^ in both seasons. Foliar application of SiNPs significantly increased leaf fresh and dry weights, with the highest improvement observed at 1200 mg L^-1^. Interaction effects showed that SiNPs mitigated salinity-induced reductions, particularly under moderate salinity (2–4 dSm^-1^), resulting in biomass comparable to non-saline plants (Tables 8 and 9). Although salinity, SiNPs, and their interaction did not significantly affect leaf dry weight during the second cut of the 2023 season (Table 9), the treatment means revealed only slight numerical variations among treatments. This result suggests that leaf dry matter accumulation was less sensitive to both salinity stress and SiNP supplementation at this stage of plant development. The maintenance of dry weight despite stress conditions may indicate an adaptive response of lemongrass plants that allowed preservation of biomass production during the later harvest, thereby reducing the magnitude of treatment-related differences.

**Table 8:** Effect of foliar application of silicon nanoparticles on leaves fresh weight (g) of *Cymbopogon citratus* plants grown under salt stress in the two consecutive seasons of 2022 and 2023

Season 2022					
First Cut					
Salinity levels (A) (dSm^-1^)	SiNPs levels (C), (mgL^-1^)				Mean
	0	400	800	1200	
Control	37.21 f	56.12 c	58.31 b	62.40 a	53.51 a
2	37.67 ef	38.07 ef	39.54 e	40.27 e	38.89 b
4	32.96 g	33.55 g	37.59 ef	47.67 d	37.94 b
6	30.30 h	33.06 g	37.39 ef	38.39 e	34.78 c
Mean	34.54 d	40.20 c	43.21 b	47.18 a	
L.S.D _0.05_	A= 1.09	B= 1.09		A×C= 2.18	
Second Cut					
Control	46.28 b	51.62 b	52.36 a	54.09 a	51.09 a
2	43.91 c	44.14 b	49.99 b	53.16 a	47.80 b
4	35.05 d	37.54 c	54.40 a	55.21 a	45.55 bc
6	36.72 c	36.88 c	43.96 c	59.51 a	44.27 c
Mean	40.49 c	42.55 c	50.18 b	55.49 a	
L.S.D _0.05_	A= 2.75	C= 2.75		A×C= 7.40	

Season 2023					
First cut					
Control	28.16 e	28.47 e	33.02 c	34.71 b	31.09 a
2	21.06 h	26.78 ef	34.54 b	36.35 a	29.68 b
4	22.42 h	26.50 f	27.76 e	31.66 c	27.08 c
6	19.58 i	24.43 g	27.84 e	30.00 d	25.46 d
Mean	22.81 d	26.55 c	30.79 b	33.18 a	
L.S.D _0.05_	A= 0.75	C= 0.75		A×C= 1.51	
Second Cut					
Control	45.17 ef	64.08 c	70.58 b	82.49 a	65.58 a
2	42.40 f	50.85 de	53.69 d	62.09 c	52.26 b
4	45.31 e	47.38 e	55.32 d	58.13 d	51.53 b
6	40.30 f	46.57 e	47.43 e	58.85 d	48.29 c
Mean	43.30 d	52.22 c	56.76 b	65.39 a	
L.S.D _0.05_	A= 2.29	C= 2.29		A×C= 5.57	

Means followed by the same letter(s)are not significantly, at 0.05 level of probability

**Table 9:** Effect of foliar application of silicon nanoparticles on leaves dry weight (g) of *Cymbopogon citratus* plants grown under salt stress in the two consecutive seasons of 2022 and 2023

**Season 2022**					
First Cut					
Salinity levels (A) (dSm^-1^)	SiNPs levels (C), (mgL^-1^)				Mean
	**0**	400	800	1200	
Control	29.65 b	44.89 a	46.64 a	49.92 a	42.77 a
2	26.36 b	26.65 b	27.68 b	28.19 b	27.22 b
4	19.77 c	20.13 c	22.55 b	28.60 b	22.76 c
6	18.18 c	19.83 c	22.43 b	23.03 b	20.86 c
Mean	23.49 b	27.87 a	29.82 a	29.93 a	
L.S.D _0.05_	A= 3.75	C= 3.75		A×C= 7.51	
Second Cut					
Control	37.02 b	41.29 b	41.88 a	43.27 a	40.86 a
2	30.73 b	30.90 b	34.99 b	37.21 b	33.45 a
4	21.03 e	22.52 e	32.64 c	33.13 c	27.33 a
6	22.03 e	22.12 e	26.38 d	35.71 b	26.55 ab
Mean	27.70	29.20	33.97	37.33	
L.S.D _0.05_	A= 15.06	C= N.S		A×C= 3.01	

Season 2023					
First Cut					
	0	400	800	1200	
Control	22.56 ab	22.78 ab	26.42 a	27.77 a	24.88 a
2	14.74 c	18.74 b	24.18 a	25.44 a	20.77 b
4	13.45 d	15.90 c	16.66 c	18.99 b	16.25 c
6	11.75 d	14.66 c	16.70 c	18.00 bc	15.27 c
Mean	15.62 d	18.02 c	20.99 a	22.55 a	
L.S.D _0.05_	A= 2.16	C= 2.16		A×C= 4.31	
Second Cut					
Control	36.14	51.26	56.46	65.99	52.46
2	29.68	35.59	37.58	43.40	36.58
4	27.18	28.43	33.19	34.88	30.90
6	24.18	27.94	28.46	35.31	28.97
Mean	29.29	35.86	38.46	44.91	
L.S.D _0.05_	A= N.S	C= N.S		A×C= N.S	

Means followed by the same letter(s) are not significantly, at 0.05 level of probability

*N.S* not significant

#### Leaf length and leaf area

Both leaf length and leaf area were significantly reduced by salinity, with maximum decreases under 6 dS m^-1^. Foliar-applied SiNPs enhanced leaf expansion, increasing length and area in all salinity treatments. The largest leaves were obtained with 1200 mg/L SiNPs, and interactions revealed that SiNPs restored leaf size under moderate salinity to levels similar to controls, demonstrating their protective effect on leaf growth (Tables 10 and 11).

**Table 10:** Effect of foliar application of silicon nanoparticles on the leaf length (cm) of *Cymbopogon citratus* plants grown under salt stress in the two consecutive seasons of 2022 and 2023

Season 2022					
First Cut					
Salinity levels (A) (dSm^-1^)	SiNPs levels (C), (mgL^-1^)				Mean
	0	400	800	1200	
Control	50.84 e	55.19 c	57.57 b	62.44 a	56.51 a
2	50.10 e	57.25 b	57.71 b	58.40 b	55.87 a
4	51.55 e	53.69 d	56.12 c	56.15 c	54.38 b
6	51.49 e	52.12 d	52.40 d	52.60 d	52.15 c
Mean	50.99 d	54.56 c	55.95 b	57.40 a	
L.S.D _0.05_	A= 0.93	C= 0.93		A×C= 1.85	
Second Cut					
Control	54.81 bc	58.92 b	60.62 ab	65.07 a	59.85 a
2	53.37 c	53.48 c	56.95 b	61.55 a	56.34 b
4	50.36 d	52.74 c	55.07 bc	64.73 a	55.72 b
6	50.62 d	52.62 c	53.62 c	54.21 c	52.77 c
Mean	52.29 b	54.44 b	56.57 b	61.39 a	
L.S.D _0.05_	A= 2.21	C= 2.21		A×C= 4.43	

Season 2023					
First Cut					
	0	400	800	1200	
Control	52.07 b	52.10 b	53.18 b	55.98 a	53.33 a
2	50.83 bc	50.85 bc	52.53 b	53.77 b	51.99 b
4	49.59 c	50.07 b	50.33 bc	52.27 b	50.56 c
6	47.51 d	49.99 c	50.44 bc	51.59 bc	49.88 c
Mean	50 .00	50.75	51.62	53.40	
L.S.D _0.05_	A= 0.97	C= N.S		A×C= 1.94	
Second Cut					
Control	66.33 b	67.44 ab	69.44 a	70.33 a	68.39 a
2	57.66 d	62.22 c	64.00 bc	64.22 c	62.02 b
4	49.78 g	52.78 ef	53.44 ef	60.33 cd	54.08 b
6	51.89 f	53.33 ef	54.11 e	56.55 de	53.97 b
Mean	56.42 c	58.94 b	60.25 b	62.86 a	
L.S.D _0.05_	A= 1.43	C= 1.43		A×C= 2.87	

Means followed by the same letter(s) are not significantly, at 0.05 level of probability

*N.S* not significant

**Table 11:** Effect of foliar application of silicon nanoparticles on the leaf area/plant (cm^2^) of *Cymbopogon citratus* plants grown under salt stress in the two consecutive seasons of 2022 and 2023

Season 2022								
First Cut								
Salinity levels (A) (dSm^-1^)	SiNPs levels (C), (mgL^−1^)							Mean
	0			400	800		1200	
Control	1626.01			1887.78	1969.70		1973.63	1864.28 a
2	1115.39			1655.44	1697.88		1992.94	1615.41 b
4	1247.99			1451.66	1587.22		1650.95	1484.46 c
6	998.44			1466.23	1510.60		1577.67	1388.24 c
Mean	1246.96 c			1615.28 b	1691.35 b		1798.80 a	
L.S.D _0.05_	A = 122.17		C = 122.17			A × C = N.S		
Second Cut								
Control	1518.48 b			1619.50 b	1889.03 a		2091.45 a	1779.62 a
2	1208.29 c			1314.44 c	1705.45 b		1886.70 a	1528.72 b
4	1097.32 c			1482.09 b	1721.66 b		1730.88 b	1507.99 b
6	1409.13 c			1426.18 b	1577.38 b		1604.52 b	1504.31 b
Mean	1308.31 b			1460.55 b	1723.38 a		1828.39 a	
L.S.D _0.05_	A = 158.56	C = 158.56				A × C = 317.08		

Season 2023								
First Cut								
	0	400			800	1200		
Control	1117.31 c	1503.74 a			1589.58 a	1625.02 a		1458.92 a
2	1240.96 b	1249.76 b			1402.78 b	1404.76 b		1324.57 b
4	1041.97 d	1104.79 c			1221.47 c	1416.14 b		1196.09 c
6	1019.57 d	1117.43 c			1201.97 c	1208.69 c		1136.92 c
Mean	1104.95 c	1243.93 b			1353.95 a	1413.65 a		
L.S.D _0.05_	A = 86.42	C = 86.42				A × C = 172.83		
Second Cut								
Control	2773.33 b	2815.65 b			2884.30 b	3727.53 a		3050.21 a
2	2060.31 c	2170.09 c			2341.61 c	2603.36 b		2293.85 b
4	1733.33 d	1879.97 d			2391.25 c	2572.25 b		2144.20 b
6	1452.60 e	1751.75 d			1767.99 d	1798.47 d		1692.70 c
Mean	2004.89 c	2154.37 c			2346.29 b	2675.40 a		
L.S.D _0.05_	A = 168.37	C = 168.37				A × C = 336.68		

Means followed by the same letter(s) are not significantly, at 0.05 level of probability

*N.S* not significant

*Overall, the* results clearly demonstrate that foliar application of SiNPs improves vegetative growth of *Cymbopogon citratus* under salinity stress. SiNPs not only increased plant height, tillering, leaf production, and biomass but also mitigated the negative effects of salt stress on leaf expansion. Interaction effects suggest that higher SiNPs doses (800–1200 mg/L) are particularly effective in restoring growth under moderate to high salinity levels, making them a promising tool for enhancing lemongrass productivity in salt-affected environments under controlled small-scale conditions. According to the results of the present study, superior vegetative growth—expressed in plant height, number of tillers, number of leaves, dry weight, leaf length, and leaf area—was achieved under non-saline conditions or at a low salinity level (2 dSm^-1^) across both experimental seasons. The enhancement in growth at lower salinity levels is logical, as increasing salinity generally exerts a progressively inhibitory effect on plant development. These findings are consistent with those reported by Parida and Das [18], who demonstrated that salt stress restricts plant growth by reducing photosynthesis and respiration, ultimately leading to diminished biomass accumulation as both salinity intensity and exposure duration increase. Similarly, Mukarram et al. [3] indicated that lemongrass, like many members of the Poaceae family, is sensitive to salinity, resulting in reduced growth under saline conditions. This observation aligns with earlier findings by Abdel Latef [19] and more recent work by Gill et al. [20]. The reduction in growth observed in the current study under higher salinity levels may be attributed to increased osmotic pressure, which lowers plant water uptake and reduces cellular hydration. Consequently, both meristematic activity and cell elongation are inhibited, as described by Munns [21]. In addition, salinity stress is known to disrupt endogenous plant hormone balance, further impairing cell division and differentiation processes [22, 23]. This disruption can limit branching and tiller formation. The significant reduction in relative water content (RWC) observed under saline conditions also supports this explanation, as decreased water availability directly restricts cell expansion and growth [24–26]. At the molecular level, salinity stress may suppress the expression of key cell cycle regulators such as cyclins and cyclin-dependent kinases, thereby reducing meristematic cell division and overall plant growth [27]. Moreover, plants exhibit different adaptive responses to salinity; some rapidly inhibit growth as a protective mechanism, while others fail to regulate growth effectively under stress, increasing their susceptibility to damage or mortality [28]. Overall, the results highlight that increasing salinity adversely affects vegetative growth through a combination of osmotic stress, hormonal imbalance, reduced water status, and impaired cellular and molecular processes.

### Effect of foliar-applied silicon nanoparticles under salinity stress on rhizome traits of *Cymbopogon citratus*

Rhizome characteristics analyzed included rhizome length, diameter, fresh weight, and dry weight (Table 12). In general, salinity stress significantly reduced all rhizome traits in both cuts and seasons, with the greatest reductions observed at 6 dSm^−1^. Foliar application of SiNPs markedly improved rhizome growth across all salinity levels. In both seasons, the highest SiNPs dose (1200 mg/L) significantly increased rhizome length, diameter, and biomass compared with the untreated control. Interaction effects revealed that SiNPs application mitigated salinity-induced reductions, particularly under moderate salinity (2–4 dSm^-1^), restoring rhizome growth to levels comparable with non-saline plants. These results indicate that SiNPs enhance underground biomass accumulation, which is critical for lemongrass regrowth and overall productivity.

**Table 12:** Analysis of variance for rhizome characters (rhizome diameter, length, fresh and dry weight of rhizome) of *Cymbopogon citratus* plants grown under salt stress in the two consecutive seasons of 2022 and 2023

S.O.V	d.f	Rhizome diameter		Rhizome length		Fresh weight rhizome		Dry weight rhizome	
		** 1 st season**	**2nd season**	** 1 st season**	**2nd season**	** 1 st season**	**2nd season**	** 1 st season**	**2nd season**
Rep	2	0.154	0.865	2.01	0.641	12.77	275.77	21.63	39.00
Salinity (A)	3	3.38**	2.26**	14.42**	3.38*	308.68**	48.91	144.74**	3.49
Silicon (C)	3	5.15**	0.289	10.49**	7.05**	34.64	31.62	14.62	4.76
A*C	9	1.66**	1.51**	8.37**	1.45	32.35	48.70	16.44*	6.77
error	30	0.088	0.247	1.19	0.821	26.87	27.51	7.22	3.11

^*^, ^**^: significant at 0.05 and 0.01 probability levels, respectively

The analysis of variance presented in the Table 11 reveals the effects of salinity (A), silicon nanoparticles (C), and their interaction (A × C) on rhizome characteristics across two growing seasons. The replication (Rep) effect was generally insignificant for most traits in both seasons, indicating good experimental uniformity. Minor variations observed, particularly in fresh and dry weights during the second season, are likely due to natural field variability rather than treatment effects. Regarding salinity (A), its effect was highly significant on most rhizome traits. Rhizome diameter and length showed highly significant differences in the first season and remained significant in the second season, although with slightly lower magnitude. Fresh weight of rhizomes was strongly affected by salinity in the first season, while its effect diminished in the second season. Similarly, dry weight showed a highly significant response in the first season but became non-significant in the second. These results indicate that salinity stress had a pronounced negative impact on rhizome growth, especially during the first season, suggesting possible seasonal differences in plant tolerance or environmental conditions. The silicon nanoparticle treatments (C) significantly influenced several rhizome traits. Rhizome diameter and length were highly significantly affected in the first season, while in the second season, silicon significantly affected rhizome length but not diameter. However, silicon application did not significantly influence fresh and dry weights in either season. This suggests that silicon nanoparticles play a more prominent role in improving structural growth traits (such as size and elongation) rather than biomass accumulation under the studied conditions. The interaction between salinity and silicon (A × C) was highly significant for most traits in the first season, including rhizome diameter, length, and dry weight, indicating that the effectiveness of silicon nanoparticles depends on the level of salinity stress. In the second season, the interaction effect remained significant for rhizome diameter but was generally reduced or non-significant for other traits. This reduction may reflect seasonal environmental variations or adaptive plant responses over time. The error values were relatively low compared to treatment mean squares, supporting the reliability and precision of the experiment.

Overall, the results demonstrate that salinity stress is a dominant factor affecting rhizome growth, while silicon nanoparticles can mitigate some of its adverse effects, particularly in improving rhizome size. The interaction effects further confirm that silicon application is more beneficial under specific salinity levels, highlighting its potential role in enhancing plant tolerance to salinity stress.

### Effect on chlorophyll content and nutrient accumulation

#### Chlorophyll value index

Salinity significantly decreased chlorophyll content in both seasons, reflecting reduced photosynthetic capacity under salt stress. Foliar-applied SiNPs enhanced chlorophyll accumulation across all treatments. The highest increase was observed at 1200 mg/L SiNPs, and interaction effects indicated that SiNPs restored chlorophyll levels under moderate salinity (2–4 dSm^-1^) to values similar to non-saline controls (Table 13).

**Table 13:** Analysis of variance for total chlorophyll content of *Cymbopogon citratus* plants grown under salt stress in the two cuts of consecutive seasons of 2022 and 2023

S.O.V	d.f	1 st season	2nd season
First cut			
Rep	2	0.928	1.55**
Salinity (A)	3	176.12**	15.09**
Silicon (C)	3	107.85**	6.18**
A*C	9	19.99*	3.28**
error	30	6.93	0.284
Second cut			
Rep	2	19.05	4.75
Salinity (A)	3	9.71*	49.13**
Silicon (C)	3	12.02*	5.62
A*C	9	9.94**	19.81**
error	30	3.14	2.49

The analysis of variance in Table 12 demonstrates the effects of salinity (A), silicon nanoparticles (C), and their interaction (A × C) on total chlorophyll content across two seasons and two cuts.

In the first cut**,** the replication (Rep) effect was non-significant in the first season but became highly significant in the second season, suggesting some environmental or experimental variability during the second season. However, the main treatments showed clear trends. Salinity (A) had a highly significant effect in both seasons, indicating that chlorophyll content is strongly influenced by salinity levels. This reflects the well-known impact of salinity stress in reducing photosynthetic pigments. Similarly**,** silicon (C) application exerted a highly significant effect in both seasons of the first cut, confirming its important role in enhancing chlorophyll content. Silicon likely improves chlorophyll stability and protects the photosynthetic apparatus under stress conditions.

The interaction (A × C) was significant in the first season and highly significant in the second season, indicating that the response of chlorophyll content to silicon depends on the salinity level. This suggests that silicon becomes more effective under certain salinity conditions, particularly in mitigating stress effects.

In the second cut**,** a slightly different pattern was observed. The replication effect remained non-significant in both seasons, indicating stable experimental conditions. Salinity showed a significant effect in the first season and a highly significant effect in the second season, again confirming its strong influence on chlorophyll content. The silicon effect was significant in the first season but became non-significant in the second season of the second cut. This may indicate that the effectiveness of silicon in enhancing chlorophyll content diminishes over time or varies with plant developmental stage. Interestingly, the interaction was highly significant in both seasons of the second cut. This highlights that even when silicon alone does not show a strong main effect, its combined effect with salinity remains crucial. It reinforces the idea that silicon primarily acts as a stress mitigator rather than a direct enhancer under non-stress conditions. The error mean squares were relatively low across all cases, indicating good experimental precision and reliability of the results.

Overall, the results suggest that salinity stress significantly affects total chlorophyll content, while silicon nanoparticles can alleviate these negative effects. The consistent significance of the interaction, especially in the second cut, emphasizes that silicon’s beneficial role is closely linked to the level of salinity stress, making it an effective tool for improving plant tolerance under saline conditions.

#### Nitrogen, phosphorus, and potassium (NPK) content

Salinity stress significantly reduced leaf N, P, and K concentrations in both seasons. SiNPs application significantly improved nutrient content, with the greatest enhancement at 1200 mg/L. Interaction analysis revealed that SiNPs mitigated nutrient deficiencies induced by salinity, particularly under 2–4 dSm^-1^, indicating improved nutrient uptake and assimilation (Table 14).

**Table 14:** Analysis of variance for nitrogen, phosphorus, potassium and proline content of *Cymbopogon citratus* plants grown under salt stress in the two consecutive seasons of 2022 and 2023

S.O.V	d.f	Nitrogen	Phosphorus	Potassium	Proline
Rep	2	0.202	0.0004	0.0269**	43.40
Salinity (A)	3	1.078**	0.007**	0.0612**	1,130,467.92**
Silicon (C)	3	0.275*	0.0063**	0.060**	159,758.23**
A*C	9	0.179*	0.0032**	0.0199**	458,721.35**
error	30	0.0712	0.0006	0.0033	4791.21

^*^, ^**^: significant at 0.05 and 0.01 probability levels, respectively

#### Proline accumulation

Proline content increased with salinity, reflecting osmotic stress. Foliar SiNPs further enhanced proline accumulation, suggesting a role in stress adaptation. The highest proline levels were recorded at 1200 mg/L SiNPs under 4–6 dSm^-1^, indicating that SiNPs further increased proline accumulation, a known osmoprotectant, suggesting improved osmotic regulation.

The analysis of variance presented in Table 14 shows the effects of salinity (A), silicon nanoparticles (C), and their interaction (A × C) on nitrogen, phosphorus, potassium, and proline contents. The replication (Rep) effect was non-significant for nitrogen, phosphorus, and proline, indicating good experimental consistency. However, it was highly significant for potassium, suggesting some variability among replicates for this trait, likely due to minor environmental differences. Salinity (A) had a highly significant effect on all studied parameters. Nitrogen, phosphorus, and potassium contents were significantly influenced, reflecting the strong impact of salinity stress on nutrient uptake and accumulation. Typically, increasing salinity reduces the availability and absorption of essential nutrients due to ionic imbalance and osmotic stress. In contrast, proline content showed a highly significant and substantial increase under salinity, which is expected since proline acts as an osmoprotectant that accumulates in plants under stress conditions to maintain cellular osmotic balance and protect cellular structures. The silicon treatment (C) significantly affected all parameters. It had a significant effect on nitrogen and highly significant effects on phosphorus, potassium, and proline. This indicates that silicon nanoparticles play an important role in improving nutrient status and enhancing stress tolerance. The increase in proline under silicon treatment suggests that silicon may enhance the plant’s defensive response mechanisms under stress conditions. The interaction between salinity and silicon (A × C) was significant for nitrogen and highly significant for phosphorus, potassium, and proline. This demonstrates that the effect of silicon nanoparticles is dependent on the salinity level. In other words, silicon is more effective under saline conditions, where it helps mitigate the adverse effects of salinity by improving nutrient uptake and further increased proline accumulation, a known osmoprotectant, suggesting improved osmotic regulation. The error values were relatively low compared to treatment mean squares, indicating a high level of precision and reliability in the experimental results.

Overall, the table clearly shows that salinity stress adversely affects nutrient content while increasing proline accumulation, and that silicon nanoparticles can alleviate these negative effects. The significant interaction between salinity and silicon further confirms that silicon application is particularly beneficial under saline conditions, improving plant nutrition and enhancing stress tolerance mechanisms.

### Effect on essential oil yield and composition

#### Essential oil percentage and yield

Salinity stress significantly decreased essential oil (EO) percentage and yield per plant in both seasons, with the largest reductions under 6 dSm^-1^. Foliar-applied SiNPs significantly increased EO content and yield. The highest EO production was obtained at 1200 mg/L SiNPs, even under moderate salinity, demonstrating the protective effect of SiNPs on secondary metabolite synthesis. The interaction between salinity and SiNPs significantly influenced essential oil (EO) percentage and EO yield (Table 15). Application of SiNPs effectively alleviated the adverse effects of salinity and enhanced EO production, resulting in values that exceeded those recorded in the untreated non-saline control. For instance, EO percentage in the first cut of 2022 increased significantly from 0.27% under the control treatment (0 dS m^-1^ salinity without SiNPs) to 0.50% under 4 dS m^-1^ salinity with SiNP application. Likewise, EO percentage increased from 0.37% to 0.43% in the second cut of 2022, from 0.49% to 0.53% in the first cut of 2023, and from 0.56% to 0.63% in the second cut of 2023. A similar pattern was observed for EO yield. In the first cut of 2022, EO yield increased significantly from 13.39 to 19.70 ml plant^-1^, while in the second cut of 2022 it increased from 13.84 to 23.05 ml plant^-1^. During 2023, EO yield increased from 12.19 to 15.73 ml plant^-1^ in the first cut and from 28.03 to 39.33 ml plant^-1^ in the second cut. These findings indicate that SiNPs markedly mitigated the negative effects of salinity and promoted essential oil biosynthesis, leading to EO percentage and yield values that were higher than those obtained under the untreated control conditions.

**Table 15:** Analysis of variance for volatile oil determination (oil% and oil yield) of *Cymbopogon citratus* plants grown under salt stress in the two consecutive seasons of 2022 and 2023

First cut					
**S.O.V**	**d.f**	**Oil percentage**		**Oil yield**	
		** 1 st season**	**2nd season**	** 1 st season**	**2nd season**
Rep	2	0.0005	0.0001	4.67	0.20
Salinity (A)	3	0.019**	0.070**	206.66**	4.72**
Silicon (C)	3	0.136**	0.022**	127.95**	29.29**
A*C	9	0.019**	0.004**	79.80**	45.79**
error	30	0.0007	0.001	2.53	1.04

**Second cut**					
Rep	2	0.0004	0.0004	2.91	5.37
Salinity (A)	3	0.0344**	0.0105**	115.08**	241.00**
Silicon (C)	3	0.0269**	0.0014*	29.84**	26.18**
A*C	9	0.0164**	0.0116**	61.48**	113.53**
error	30	0.0004	0.00036	3.22	4.52

^*^, **: significant at 0.05 and 0.01 probability levels, respectively

The analysis of variance in Table 15 clearly demonstrates the influence of salinity (A), silicon nanoparticles (C), and their interaction (A × C) on volatile oil percentage and oil yield across both seasons and cuts. In the first cut**,** the replication (Rep) effect was non-significant for both oil percentage and oil yield in the two seasons, indicating good experimental precision and uniformity. Salinity (A) had a highly significant effect on both oil percentage and oil yield in both seasons. This suggests that salinity stress markedly alters secondary metabolism, often leading to changes in essential oil accumulation. In many aromatic plants, moderate salinity can stimulate oil biosynthesis, while higher salinity may reduce yield due to growth inhibition. The silicon treatment (C) showed highly significant effects on oil percentage and oil yield in both seasons. This indicates that silicon nanoparticles play a strong role in enhancing essential oil production, possibly through improving plant physiological performance, photosynthesis, and stress tolerance. The interaction (A × C) was also highly significant for all traits, confirming that the response of oil percentage and yield to silicon depends on salinity levels. This interaction highlights that silicon is particularly effective under stress conditions, where it helps plants maintain metabolic activity and improve oil productivity. In the second cut**,** a similar trend was observed. The replication effect remained non-significant, supporting the reliability of the experiment. Salinity (A) continued to show highly significant effects on both oil percentage and oil yield in both seasons, with even greater mean square values for oil yield in the second season, indicating a strong influence of salinity on oil productivity at later growth stages. The silicon effect (C) was highly significant for oil yield in both seasons and for oil percentage in the first season, while it showed a significant effect in the second season. This suggests that silicon consistently enhances oil yield, although its effect on oil concentration may vary slightly between seasons. The interaction (A × C) remained highly significant for all parameters, reinforcing that silicon application is most beneficial under specific salinity conditions and plays a key role in mitigating stress effects while enhancing secondary metabolite production. The error mean squares were low relative to treatment effects, indicating high experimental accuracy. Overall, the results indicate that salinity significantly affects volatile oil production, while silicon nanoparticles enhance both oil percentage and yield. The strong and consistent interaction effect confirms that silicon is particularly effective in alleviating salinity stress and improving essential oil productivity.

These findings are in agreement with previous studies, such as Taiz et al., [29], who reported that environmental stresses like salinity can significantly influence secondary metabolite production, including essential oils. Additionally, Epstein [30] highlighted the beneficial role of silicon in improving plant tolerance to abiotic stress. Similar results were also reported by Irfan et al. [31], who found that silicon application enhanced essential oil yield under salinity conditions by improving physiological and biochemical processes.

#### Essential oil composition (GC–MS analysis)

**Fig. 3:**
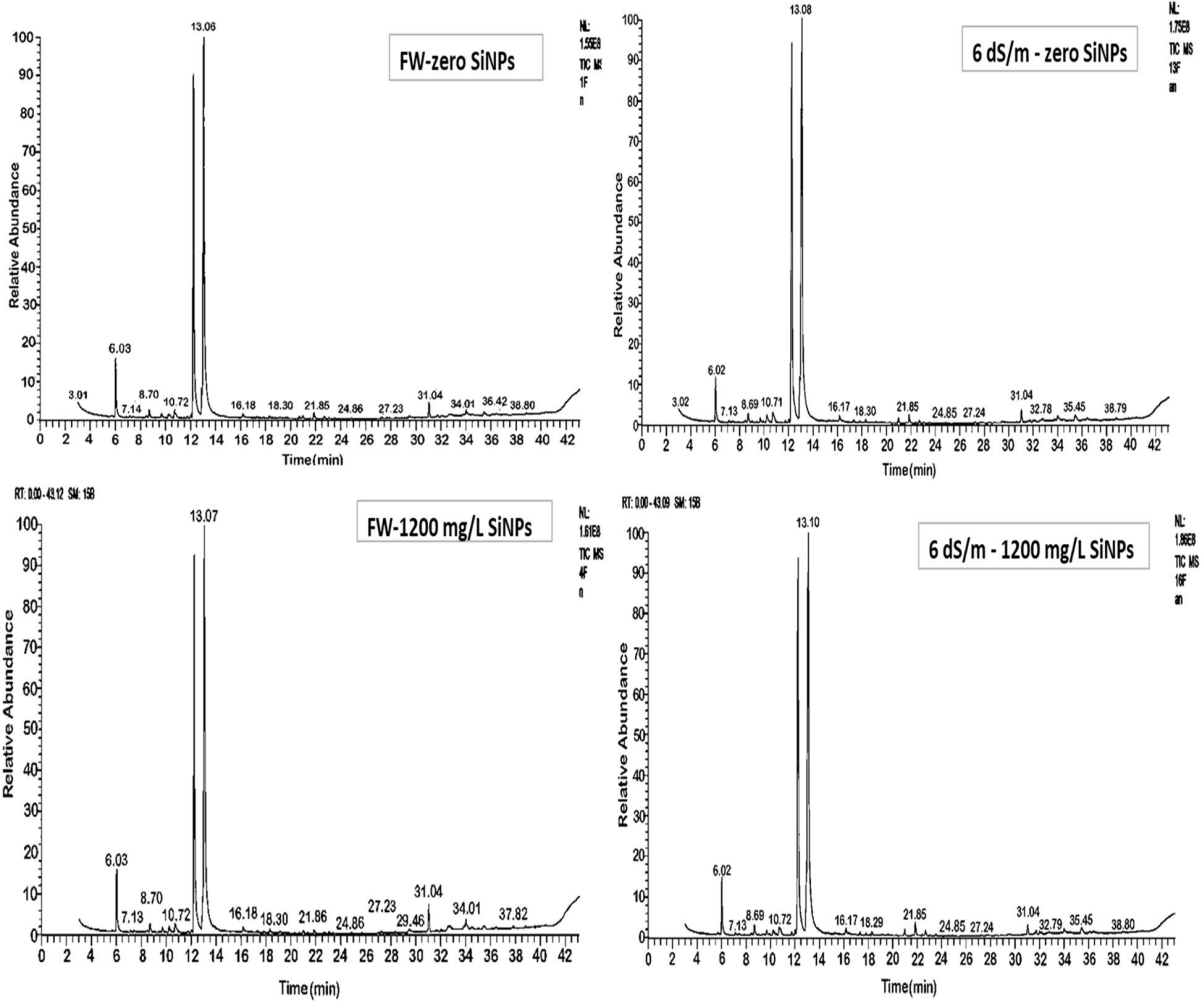
GC of essential; oil components in lemongrass plants treated with fresh water and 6 dSm^−1^ at zero and 1200 mg/L SiNPs foliar application

GC–MS analysis (Fig. 3) revealed that salinity stress altered the relative composition of major essential oil (EO) constituents. However, foliar application of silicon nanoparticles (SiNPs) stabilized EO composition, maintaining higher levels of key bioactive compounds such as citral, neral, and geranial even under moderate salinity stress. Also, GC–MS analysis identified 48 chemical constituents in the essential oil of Cymbopogon citratus. The major compounds detected were citral (geranial + neral), neral, β-myrcene, nerol, limonene oxide, linalool, myrtenol, and geraniol acetate. The quality of lemongrass essential oil is primarily determined by its citral content, which consists of a mixture of the isomers geranial and neral. Citral was the predominant constituent in all treatments, accounting for 45.47–49.40% of the total oil composition. The highest citral content (49.40%) was recorded in plants treated with 800 mgL^-1^ SiNPs under 6 dS m^-1^ salinity stress, followed by 48.91% in plants treated with 400 mgL^-1^ SiNPs under non-saline conditions. When geranial and neral contents were combined, the total citral concentration exceeded the minimum requirement of 75% specified by ISO 3217 (1974) for Cymbopogon citratus essential oil. Neral (β-citral) was the second most abundant component, ranging from 36.07 to 40.62%. The highest neral content (40.62%) was observed in plants grown under 2 dS m^-1^ salinity without SiNP application, followed closely by 40.28% in plants treated with 400 mgL^-1^ SiNPs under the same salinity level.

The concentration of β-myrcene varied between 3.11 and 6.30%. The maximum β-myrcene content (6.30%) was obtained with 400 mgL^-1^ SiNPs under 2 dS m^-1^ salinity, followed by 6.24% under 4 dS m^-1^ salinity with the same SiNP concentration. Nerol content ranged from 0.75 to 2.55%, with the highest value recorded in plants treated with 800 mgL^-1^ SiNPs under 4 dS m^-1^ salinity stress. The second highest value (2.38%) was obtained with 1200 mg L^-1^ SiNPs under the same salinity level. Linalool represented a minor component of the oil, ranging from 0.81 to 1.05%. The highest linalool concentration was observed in plants treated with 400 mg L^-1^ SiNPs under non-saline conditions, followed by plants receiving the same SiNP concentration under 2 dS m^-1^ salinity. Myrtenol (previously referred to as myrtanal) ranged from 0.30 to 0.96%. The highest concentration was recorded in plants treated with 1200 mgL^-1^ SiNPs under 2 dS m^-1^ salinity stress, followed by plants receiving 800 mgL^-1^ SiNPs at the same salinity level. Geraniol acetate content ranged from 0.45 to 0.77%. The highest concentration (0.77%) was detected in plants treated with 800 mg L^-1^ SiNPs under 6 dS m^-1^ salinity stress, followed by 0.75% in plants exposed to 6 dSm^-1^ salinity without SiNP sapplication. Overall, the GC–MS results indicate that both salinity and SiNP treatments influenced the relative abundance of major essential oil constituents, with moderate SiNP application rates generally enhancing the accumulation of desirable aroma compounds, particularly citral and its related monoterpenes.

These findings are consistent with the results of Mukarram et al. [3], who reported that SiNPs preserved essential oil composition in lemongrass under saline conditions, and Aloufi et al. [32], who demonstrated that nanoparticle applications enhance the accumulation and stability of major EO constituents under abiotic stress. Moreover, foliar application of SiNPs significantly enhanced vegetative and rhizome growth, improved nutrient status, promoted osmotic adjustment, and increased both EO yield and quality in *Cymbopogon citratus* under salinity stress. These improvements are in agreement with Wang et al. [33], who highlighted the role of SiNPs in improving plant growth and nutrient uptake under stress conditions, as well as Khan et al. [34], who emphasized the importance of silicon in enhancing osmotic regulation and stress tolerance. Additionally, the overall mitigation of salinity stress and enhancement of plant performance observed in this study are supported by Akhter et al., [35], who reported that silicon improves physiological and biochemical responses under saline environments.

The observed dose-dependent response, with 800–1200 mgL^−1^SiNPs being the most effective—particularly under moderate salinity levels (2–4 dSm^-1^)—further confirms that silicon nanoparticles represent a promising nanotechnology-based strategy for alleviating salinity stress and enhancing both the productivity and essential oil.

Overall, for summarization, all previous results arepresented in Table 16 that clearly demonstrate that increasing salinity levels adversely affected all vegetative, rhizome, physiological, and biochemical traits of *Cymbopogon citratus.* The negative impact was most pronounced at the highest salinity level (6 dSm^-1^), confirming the inhibitory effects of salinity on plant growth and metabolism, as reported by Munns and Tester [36, 37]. In contrast, foliar application of silicon nanoparticles (SiNPs) consistently improved all studied traits in a dose-dependent manner, with the highest concentration (1200 mg/L) showing the greatest enhancement, particularly under moderate stress conditions (2–4 dSm^-1^). Moreover, the interaction between salinity and SiNPs was significant for most traits, indicating that the beneficial effects of SiNPs are strongly dependent on stress intensity.

**Table 16:** Summary of the Effects of Foliar-Applied SiNPs on *Cymbopogon citratus* grown under Salt Stress

Trait	Salinity Effect	SiNPs Effect	Salinity × SiNPs Interaction	Optimal Treatment
Plant Height (cm)	Decreased with increasing salinity; lowest at 6 dS m^-1^	Increased with increasing SiNP dose; highest at 1200 mg L^-1^	SiNPs mitigated salinity reductions; 1200 mg/L under 4 dS m^-1^ was stronger than 2 dS m^-1^	1200 mgL^−1^SiNP + 4 dS m^-1^ was stronger than 2 dS m^-1^
Number of Tillers/plant	Reduced under higher salinity	Enhanced with SiNPs	High SiNP doses restored tillering under moderate salinity	1200 mgL^−1^SiNP + 4 dS m^-1^ was stronger than 2 dS m^-1^
Number of Leaves/plant	Declined with salinity stress	Increased with SiNPs	800–1200 mg/L SiNPs restored leaf number under moderate salinity	1200 mgL^−1^SiNP + 4 dS m^-1^ was stronger than 2 dS m^-1^
Leaf Fresh Weight (g)	Decreased under salinity	Increased with SiNPs	SiNPs partially restored fresh biomass under moderate salinity	1200 mgL^−1^SiNP + 4 dS m^-1^ was stronger than 2 dS m^-1^
Leaf Dry Weight (g)	Slightly reduced by salinity	Slight increase with SiNPs	Interaction less pronounced in second season	1200 mgL^−1^ SiNP + 4 dS m^-1^ was stronger than 2 dS m^-1^
Leaf Length (cm) & Leaf Area (cm^2^)	Reduced by salinity	Increased with SiNPs	SiNPs restored leaf size and area under moderate salinity	1200 mgL^−1^SiNP + 4 dS m^-1^ was stronger than 2 dS m^-1^
Rhizome Length (cm)	Decreased under salinity	Increased with SiNPs	Moderate-high SiNPs restored length under stress	1200 mgL^−1^SiNP + 4 dS m^-1^ was stronger than 2 dS m^-1^
Rhizome Diameter (mm)	Reduced by salinity	Increased with SiNPs	High SiNP doses mitigated salinity effect	1200 mgL^−1^SiNP + 4 dS m^-1^ was stronger than 2 dS m^-1^
Rhizome Fresh & Dry Weight (g)	Decreased under salinity	Increased with SiNPs	Interaction significant; biomass restored under moderate salinity	1200 mgL^−1^SiNP + 4 dS m^-1^ was stronger than 2 dS m^-1^
Chlorophyll Value Index (SPAD)	Reduced under salinity	Increased with SiNPs	Moderate-high SiNP doses restored chlorophyll	1200 mgL^−1^SiNP + 4 dS m^-1^ was stronger than 2 dS m^-1^
N, P, K Content (%)	Decreased under salinity	Increased with SiNPs	Interaction significant; SiNPs enhanced nutrient uptake under stress	1200 mgL^−1^SiNP + 4 dS m^-1^ was stronger than 2 dS m^-1^
Proline (µg/g)	Increased under salinity	Further enhanced by SiNPs	Highest proline at high SiNP doses under moderate-high salinity	1200 mgL^−1^SiNP + 4 dS m^-1^ was stronger than 2 dS m^-1^
Essential Oil %	Decreased with salinity	Increased with SiNPs	SiNPs restored EO % under moderate salinity	1200 mgL^−1^ SiNP + 4 dS m^-1^ was stronger than 2 dS m^-1^
EO Yield (ml/plant)	Reduced under salinity	Increased with SiNPs	SiNPs restored EO yield under moderate salinity	1200 mgL^−1^SiNP + 4 dS m^-1^
EO Composition (citral, neral, geranial)	Altered under salinity	Maintained or improved with SiNPs	SiNPs stabilized EO quality under stress	1200 mgL^−1^SiNP + 4 dS m^-1^ was stronger than 2 dS m^-1^

Salinity levels: 0 (control), 2, 4, 6 dS m^-1^; SiNP doses: 0, 400, 800, 1200 mgL^-1^; Optimal treatments indicate combinations that consistently restored growth, biochemical, and essential oil traits close to non-saline control levels

## Conclusion

The results of the present study clearly demonstrate that salinity stress significantly impairs the growth, physiological performance, nutrient status, and essential oil productivity of lemongrass plants, particularly at higher salinity levels (4–6 dSm^-1^). In contrast, optimal vegetative growth, rhizome development, and oil yield were achieved under non-saline and low salinity conditions (0 and 2 dSm^-1^). The adverse effects of salinity were mainly associated with osmotic stress, ion toxicity, nutrient imbalance, reduced photosynthetic efficiency, and disturbances in metabolic processes. The application of silicon nanoparticles (SiNPs), especially at higher concentrations (3000 mgL^-1^ as soil application and 1200 mg L^-1^ as foliar spray), proved highly effective in mitigating the negative impacts of salinity. SiNPs enhanced plant growth, improved nutrient uptake (N, P, and K), maintained better water status, increased chlorophyll content, and stimulated antioxidant defense mechanisms. Additionally, SiNPs contributed to improving both the yield and composition of essential oils, even under saline conditions. Overall, the integration of silicon nanoparticles represents a promising technique for enhancing lemongrass tolerance to salinity stress, improving productivity, and maintaining essential oil quality. These findings highlight the potential application of SiNPs in sustainable agricultural practices, particularly in salt-affected soils under pot experiment conditions which may not fully represent field environments and their associated variability. Future field-based studies and molecular analyses are needed to further validate and explain the observed responses under salinity stress.

## Data Availability

Availability of data and materials: The raw data supporting the conclusions of this article will be made available by the authors on request.

## Abbreviation

SiNPs: Silicon nanoparticles
